# Synchrotron tomography applications in agriculture and food sciences research: a review

**DOI:** 10.1186/s13007-022-00932-9

**Published:** 2022-08-13

**Authors:** Navnath S. Indore, Chithra Karunakaran, Digvir S. Jayas

**Affiliations:** 1 Biosystem Engineering, University of Manitoba, Winnipeg, MB R3T 5V6 Canada; 2 Canadian Light Source Inc., Saskatoon, SK S7N 2V3 Canada

**Keywords:** Synchrotron X-ray, X-ray absorption, X-ray phase-contrast, Microcomputed tomography, Plant imaging, Seed imaging, Soil-root medium imaging, Food imaging

## Abstract

Synchrotron imaging is widely used for research in many scientific disciplines. This article introduces the characteristics of synchrotron X-ray imaging and its applications in agriculture and food science research. The agriculture and food sector are a vast area that comprises of plants, seeds, animals, food and their products; soils with thriving microbial communities; and natural resources such as water, fertilizers, and organic matter. These entities have unique internal features, structures and compositions which differentiate them from each other in varieties, species, grades, and types. The use of a bright and tuneable monochromatic source of synchrotron imaging techniques enables researchers to study the internal features and compositions of plants, seeds, soil and food in a quick and non-destructive way to enhance their use, conservation and productivity. Synchrotron’s different X-ray imaging techniques offer a wide domain of applications, which make them perfect to enhance the understanding of structures of raw and processed food products to promote food safety and security. Therefore, this paper summarizes the results of major experiments carried out with seeds, plants, soil, food and relevant areas of agricultural sciences with more emphasis on two synchrotron X-ray imaging techniques: absorption and phase-contrast imaging and computed tomography.

## Introduction

Agriculture has made many advancements after the nineteenth century to ensure food security for the world’s ever-increasing population with the help of high-yielding varieties, integrated farming, post-harvest management, mechanization, and sustainable use of natural resources. Agriculture inputs (seeds, soil, and fertilizers) and produced food (plants, fruits, grains have unique internal features, structures, and compositions that differentiate them from each other in varieties, species, grades, and types. Every entity of agriculture is adapted and suited to local conditions by altering their internal structures, composition, and features, either naturally or by the intervention of human skills. The study of these internal features at the micro to nanoscale level is vital to study species and composition for addressing several questions. Therefore, many studies have been carried out for decades using tools like optical light microscopy, transmission electron microscopy, scanning electron microscopy, and histology to gather vital microscopic information. Although these techniques were able to provide information, they had certain limitations like destructive sample preparation, sampling area, accuracy of sample location, less number of samples, and extensive sample preparation protocols, all of which can make data interpretation more difficult [1]. Even methods like histology cannot give reliable visualization of small cellular interspaces or indicate the existence of networks of spaces [2]. Therefore, to overcome these challenges, the applications of X-ray imaging have been started since 1980 in agriculture and food sciences with applications in cotton [3], lettuce [4], pine [5], tomato [6], rice [7], wheat [8, 9], pecan [10], fruits [11–13], plant leaves [14], and soils[15]. A detailed review was carried out on the use of X-ray computed tomography (CT) in agriculture and food applications [16] with recommendations on the requirement of more advanced X-ray techniques and applications in these areas and the development of commodity-specific algorithms for image processing like those found in the medical imaging field. These conventional X-ray CT systems have certain limitations like low resolution, more scanning time, beam hardening, low energy or photon flux, and nonselective wavelength. Therefore, synchrotron X-ray imaging techniques have gained pace in the last two decades and researchers across the globe have started their use in studies such as: 1. dynamic processes like water or nutrient flow in live plants [1, 14, 17–25], internal structural information in seeds [2, 26–30], fruits [13, 19], wood [23, 31–33], and soil root medium [24, 34–43]; 2. development of innovative nutritional foods [44–49], and 3. disease management in plants and animals [50–53]. These studies have been carried out using non-invasive synchrotron X-ray imaging methods and are summarized in this article. There are a few questions that require answering on what made synchrotron a popular tool among agriculture and food scientists. This article highlights the unique capabilities of two synchrotron X-ray techniques; absorption micro-CT and phase contrast micro-CT over conventional X-ray imaging methods used in agriculture and food sciences. Paper has also summarized all synchrotron machine parameters used till date in agriculture and food science studies, which will be very useful in planning of new experiment.

### Synchrotron X-rays

A unique electromagnetic spectrum is created by bending the path of electrons traveling at the speed of light in a synchrotron [54]. During this process, synchronous addition of energy to the electron beam in the ring takes place by changing the energy of electrons or adding energy to the beam without changing the orbit of electrons. This powerful scientific tool was invented back in the late 1960s, and the first-generation multi-GeV storage ring became operational in 1974 at Stanford Synchrotron Laboratory [55]. The synchrotron (SR) generation facility can be very large, the small one equivalent to a football field size. To date, around 67 synchrotron facilities are operational, among the 86 established synchrotron facilities across the world [56] and these establishments themselves explain the importance of technology in research and development across the world.

**Fig. 1:**
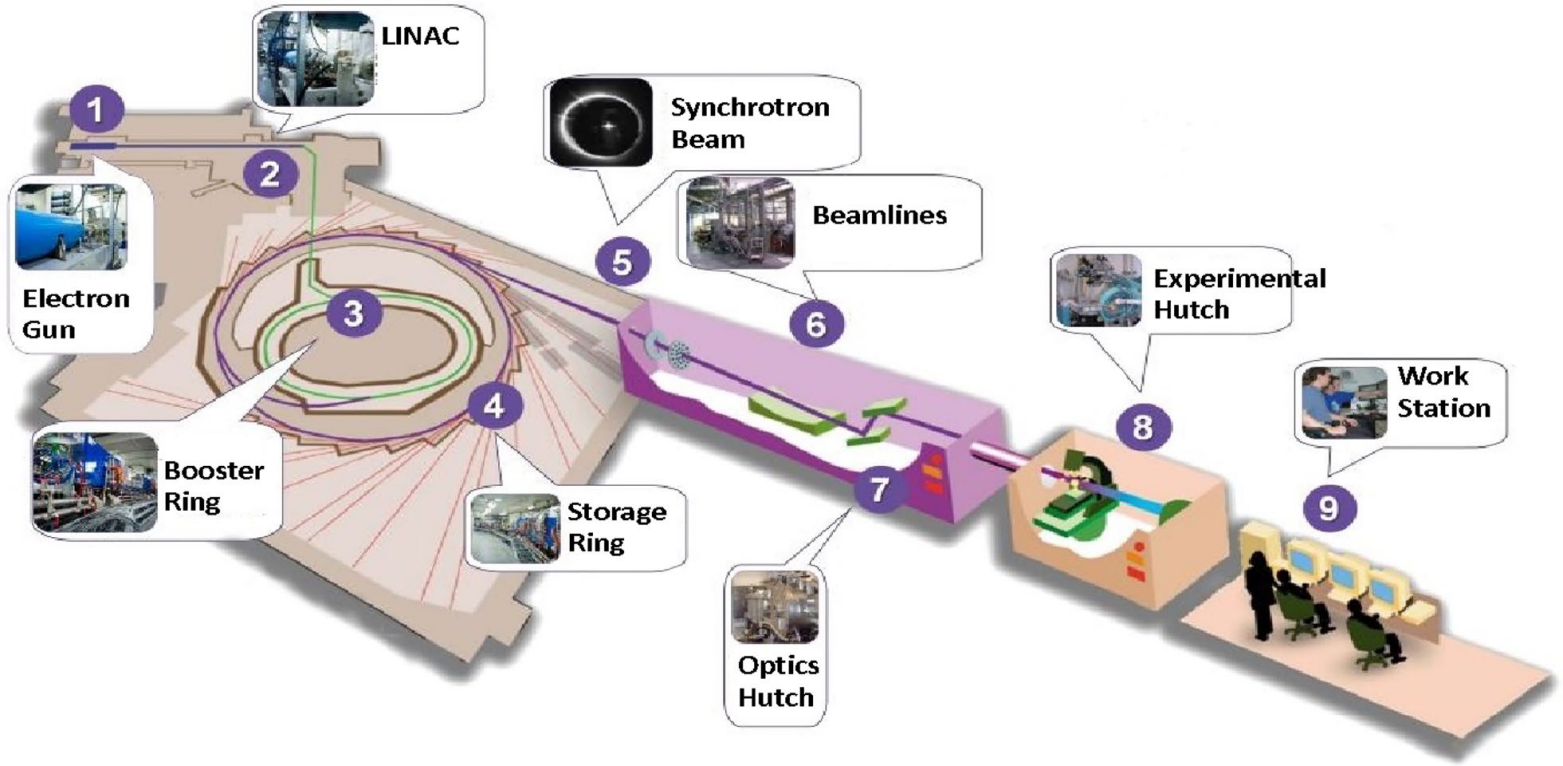
Major components of a synchrotron X-ray imaging beamline system (Source: Canadian Light Source, Saskatoon; www.lightsource.ca)

The facility of the Canadian Light Source (CLS), Saskatoon is taken as an example to briefly describe the layout of a synchrotron imaging facility (Fig. 1): it consists of 1. an electron gun, 2. linear accelerator, 3. booster ring, 4. high vacuum storage ring, 5. insertion devices (such as bending magnets, wigglers, and undulators), 6. beamlines with monochromators, 7. optical hutch (slits, filters, mirrors), 8. experimentation hutch (sample loading), and 9. workstations (data acquisition and processing).

### Properties of synchrotron light and interaction

The X-rays generated from synchrotrons generally are of three types: soft, tender, and hard. X-rays are categorized as soft X-rays when generated at or less than 2 keV, tender X-rays at 2–6 keV energy, and hard X-rays at greater than 6 keV [57]. Synchrotron X-rays have unique properties such as high flux density, coherent, and selective wavelength/monochromatic. The developed synchrotron facilities around the world have ring energy (1.7–9.0 GeV), ring current (0.010–0.5 A), ring size (198–2304 m), emittance (1 nm radx 10 pm rad to 6 nm rad × 100 nm rad) and brilliance (10^18^–10^23^ ${\mathrm{ph}.\mathrm{s}}^{-1}{{\mathrm{mm}}^{-2}\mathrm{mr}}^{-2}{0.1\%\mathrm{bw}}^{-1}$) [58, 59]. More details about the brilliance for other cases could be found elsewhere [59, 60]. This paper has covered absorption and phase-contrast synchrotron imaging techniques, where SR X-ray is attenuated by photo absorption and elastic (Thomson) scattering processes and their detailed interactions with the material can be found in the literature [59, 61, 62].

Synchrotron imaging methods are used either in two-dimensional (2D) or three-dimensional (3D) imaging. The tomography and microcomputed tomography are the focus of this review paper. In 2D imaging, a set of two-dimensional images are acquired using a monochromatic source just like radiographs, hence the beam-hardening issues are avoided that are more common in polychromatic beam [63]. The contrast in images is driven by a combination of absorption or density differences, and phase or small density variations of the sample components. The 3D computed tomography makes use of computer-processed combinations of many X-ray projections (2D images or radiographs) taken from different angles of a sample in absorption or phase contrast imaging, then produces cross-sectional (tomographic) images of specific areas of the sample.

### Synchrotron absorption tomography or micro-computed tomography (SR-XTM or SR-μCT)

**Fig. 2:**
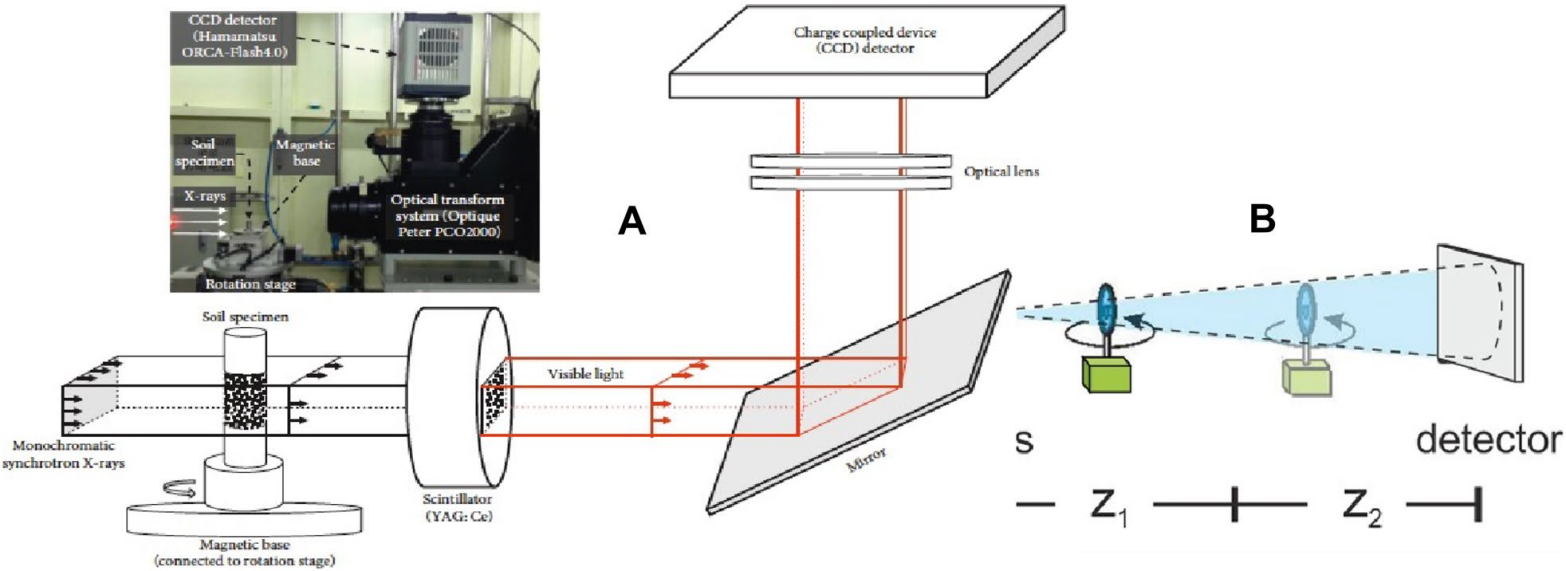
**A** Basic layout of synchrotron X-ray micro-CT [34] and **B** Setup of phase contrast micro-CT where sample to detector distance is adjustable [36, 66]

The general procedure of imaging in SRXTM or μCT consists of (Fig. 2): 1. selection of photon energy and flux of desired wavelength by use of a monochromator, 2. sample rotation about an axis from 0 to 180° in different step sizes in CT, 3. acquisition of images by a set of detectors, and 4. image processing and segmentation. The contrast in imaging is driven by absorption differences and density differences. There is no beam-hardening with this technique, which causes an inaccurate portrayal of the true X-ray absorption of the specimen and inaccurate measurements, and digital sections are more difficult to segment in image processing [64]. Synchrotron X-ray tomography has proven to be an excellent imaging system for the study of the fossil as well as modern plants [19]. The SR-µCT analysis is based on transmission images after the material attenuates the incoming X-ray beam either by absorption or by scattering (Eq. 1). The correlation between the incident or transmitted beam intensity and the material properties can be related through the Lambert–Beer law [31, 65]:

1 $$I={I}_{0}{e}^{-\mu .t},$$

where I is transmitted beam, I_0_ is incident beam, µ is linear absorption coefficient, and t is the material thickness [58].

### Synchrotron phase-contrast imaging or microtomography (SR-PCI or SRP-μCT)

**Fig. 3:**
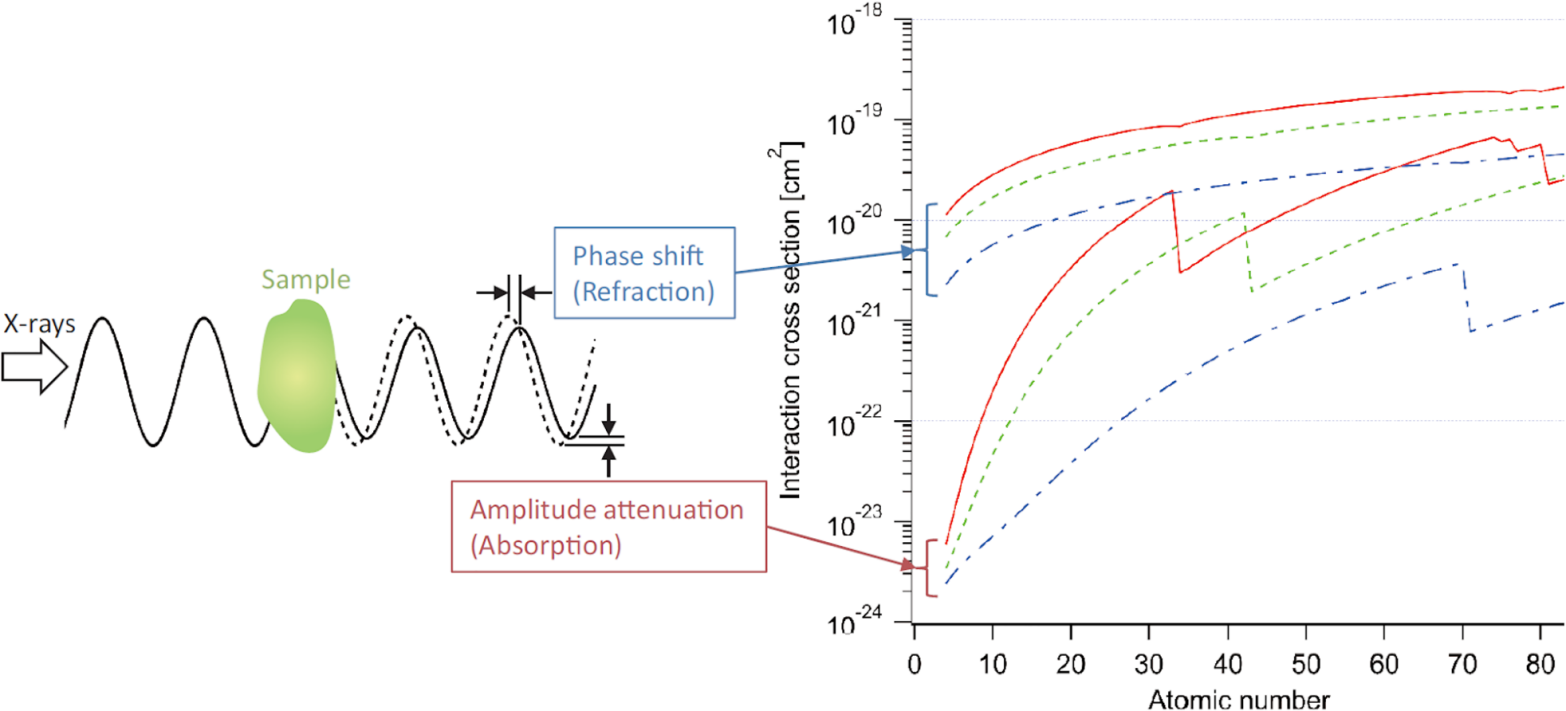
Schematic diagram of how X-rays pass through object. Dashed line shows X-ray wave without object, and solid with object. Upper curves: cross section corresponding to X-ray phase shift. Lower curves: cross section corresponding to X-ray absorption [72]

The imaging setup is very similar to SR-absorption based μCT imaging, the only difference is that rather than placing the detector close to the sample, it is located at some variable distance which gives rise to Fresnel fringes [67] (Fig. 3). Sample to detector distance is set in a such a way that it always less than sample to source distance [53]. The technique makes use of the refraction of X-rays by the sample and highlights the edges and internal boundaries of a sample, therefore, low-density materials (soft plant parts) can also be imaged, which do not absorb X-rays sufficiently to form a distinct absorption X-ray image [68]. Phase-contrast imaging method is most sensitive to small changes in the refractive index (Eq. 2, 3), which creates edge enhancement and increases the contrast of the edges of structures or material boundaries [69]. Fresnel diffraction, resulting from an adjustment of distance, allows for control of the edge enhancement effect which is the key feature of phase-contrast imaging [1, 70, 71]. It is a powerful tool to obtain quantitative data from biological samples because of the significant difference in electron density between air, biological tissues, and water [25].

The refractive index of matter can be defined as [53],

2 $$n=1-\updelta +i\beta ,$$

where δ is the decrement in part of the refractive index, which is the phase shift term, and β is the attenuation term for absorption.

The total phase shift ∅ can be related to δ as, ∅ = δkz and the absorption term β can be related to k and z as

3 $${\mu }^{\mathrm{^{\prime}}}\hspace{0.17em}=\hspace{0.17em}\mu .t\hspace{0.17em}=\hspace{0.17em}2\beta k.t,$$

where μ is the absorption coefficient, k is the wavenumber, and t is the object thickness. The detailed theory about SR-μCT and SRP-μCT concepts, working and synchrotron interactions is available [53, 60]. A comparison in working principles of both absorption and phase contrast imaging is illustrated in Fig. 3.

### Image processing used in agriculture and food sciences studies

**Fig. 4:**
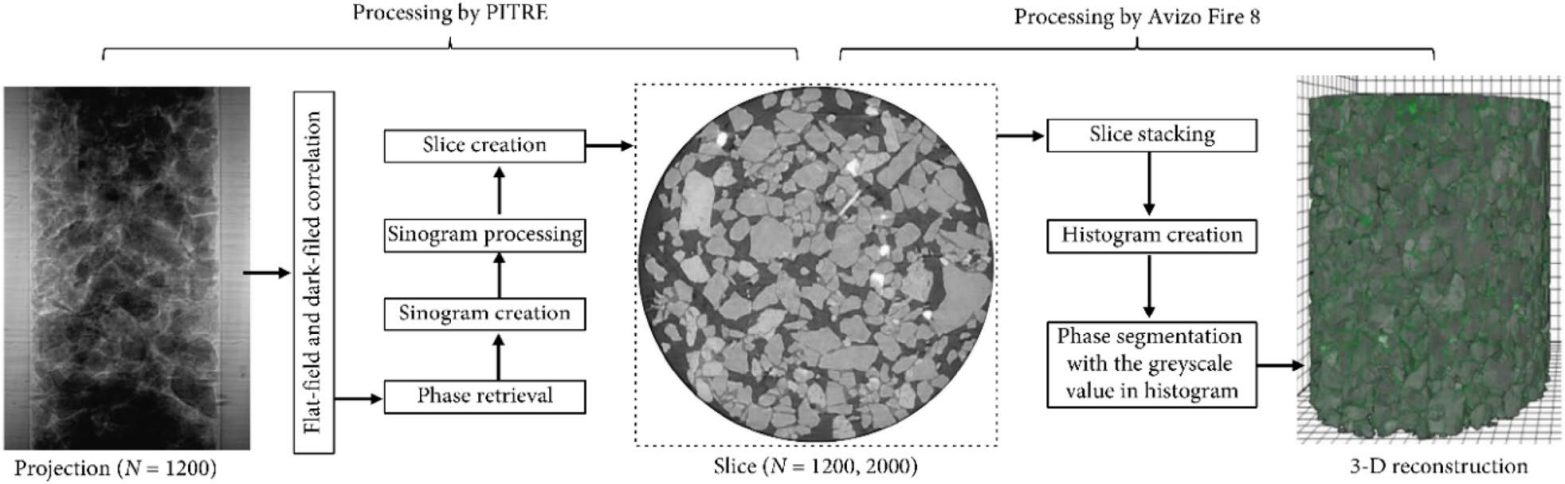
Phase retrieval, slice creation, and 3D reconstruction of a soil sample [36]

It is an important step in the analysis of acquired data which is in pixel form with associated grayscale value. In computed tomography, grayscale values represent the X-ray absorption/phase values, where higher (brighter) grayscale values indicate higher absorption and lower (darker) indicate lower absorption [54]. The acquired raw projections in both mentioned imaging methods are sometimes noisy and associated with artifacts like rings, and motion streaks, which can be caused due to motion, imaging system, and sample conditions. These artifacts can be removed by reconstruction of images, which can be achieved by dedicated software like PITRE, UFO-KIT, Recon, PyHST, Octopus 8.3, SYRMEP TomoProject, VG Studio Max, and DAWN [1, 23, 34, 66, 70, 73–77]. Initial normalization can be done by subtracting flat (images taken with a beam and without sample) and dark (images taken with beam off) images from radiographs (Fig. 4) by any software like PITRE [70]. Preliminary reconstruction of tomographic images is done using filter back projections and non-iterative phase retrieval algorithms (like Paganin) to produce images with less noise and artifacts [16]. Reviewed studies have used modern approaches like Fourier transform filtered backprojection [2], correction of sinograms, phase retrieval and ring removal by filters and improved signal-to-noise ratio [66, 73]. The reconstructed data are then further processed for improving contrast, segmentation and thresholding by either one of the software; ImageJ plugins (Polar transformer) or AVIZO [21, 23, 39, 74] or Dragonfly [78], Pore3D [47], 3dma, and customized Matlab program [77]. It is important to select X-ray energy that is appropriate for the material under testing to optimize both spatial resolution and contrast sensitivity, so an adequate signal-to-noise ratio could be obtained [42]. The sequence of steps involved in the reconstruction and processing of images in detail is discussed (Fig. 4) as an example here, starting from flat and dark field correction, reconstruction of acquired projections by PITRE then to segmentation and thresholding in 3D by Avizo [36]. Applications of advanced approaches like machine learning to acquired data [39, 79] and deep learning for phase retrieval [80, 81] are limited (only a few like local thickness analysis for pore space characterization [38]) in reviewed studies, which means conducted studies were not able to harness the full potential of synchrotron imaging unlike in material science and medical imaging. It has been found that most of the studies processed 8-bit datasets except in a few studies where (16-bit scale of 0–65,535) datasets were used [53] and one group has used the statistical tool of fisher ratio index for enhancing contrast in reconstructed images [26].

### Applications in agriculture

Synchrotron X-ray imaging is already a well-established technique and has many applications in medical and materials sciences. It has been developed for several decades but only in the last two decades, SR has been used in plant science experiments [82, 83]. Published research has demonstrated that SR-μCT and SRP-μCT were extensively used for creating three-dimensional images which have enabled researchers to look at different virtual slices of samples and visualize internal micro-structures of plants, soil, and seeds without sample destruction [26].

### Seeds

Seeds are an integral part of the plant ecosystem which store vital information, food, and energy. The study of modern and ancient seed structures at micro-level is important to conduct their evaluation for vigor, dormancy and other characteristics. Seed growth and physiology are actually dependent on several microscopic features, including cell shape, cellular water potential and the presence of intercellular voids [78]. The structure and composition of cell corners in a dry seed are tissue-dependent and vary with plant development [2]. Synchrotron X-ray imaging is a perfect tool for studying these details in seeds at a cellular level, hence it was used to find links between modern and fossil seeds [19], seed coat thinning [29], developmental stages during germination [26], intercellular void network [2, 13]. Paleobotanical studies depend on the recognition and accurate identification of extinct species of plants. The archaeological samples are precious and require non-invasive approach. Traditionally, histology was the only method which involved differential staining to reveal differential tissue and cell wall chemistry, but with synchrotron, differences can be highlighted by varying X-ray absorption. Therefore, Smith et al. [19] carried out successful digital visualization of different components of modern and fossil fruit seeds (Table 1) and visualized chemically distinct materials of preserved specimens differentially because of a difference in X-ray absorption values. Traditional sectioning does not infiltrate well due to hardness of herbarium specimens, but it can be addressed by digital segmentation and dissection of data in 3D of synchrotron X-ray images for creating virtual infills for simulating taphonomic effects [19]. A similar study was reported dealing with the study of ancient seed grains [29] and documented the evidence of seed coat thinning (from 23.2 to 10.6 μm) in horse gram seed of archaeological and modern seed samples (Table 1). Synchrotron was used extensively for fossil plants, cretaceous flowers [75], and seeds imaging and contributed dramatically to expanding the level of information available for studying diverse fossil plants [84].

**Table 1:** Synchrotron imaging operation parameters used in agriculture and food science research

Technique	Material	Purpose/findings	Beamline and beam size (H, mm × V, mm)	Energy (keV)	Resolution (μm)	No of Projection or Increment angle (°)	Exposure per image/total time (s/min)	Field of view mm × mm	Detector/detector pixel	Voxel (mm/μm)	Source to detector(Sr/d) and sample to detector distance (Sl/d), cm	References
Plants and fruits												
SR-XTM	Modern and Fossil plants	Internal structure	TOMCAT, Swiss (50 × 4)	9.9	0.35	1500	0.42/10.5	1 × 1.4	2560 × 2160	400 × 400 × 400 μm	100(Sl/d)	[19]
SRPC-μCT	Canola plant	Water and nutrient transport	BMIT-Canadian light source (40 × 5)	18, 24	4.3	1800, 5001	1/83	4 × 10	4000 × 2600	0.7 × 0.7 × 0.7 mm	80, 60(Sl/d)	[1]
SR-μCT				18	8.75	1800	2.3/69				8.5(Sl/d)	
SR-PCI	Wheat	Fusarium disease detection		18	8.75	*–	1 s/–		4000 × 2600	1 × 1 × 1 mm	80(Sl/d)	[90]
SRP-TM	Live vines of plant	Plant xylem network	ALS, Berkeley CA USA (40 × 4.6)	10–18	4.5	720/0.25°	0.1–1/40	8.3 × 18	4006 × 2672	5 × 5 × 5 μm	1000 (Sr/d)	[23]
SR-μCT	Rose peduncles	Effect of SR- X-ray and residence time on quality	BESSY II, Germany (200 × 100) μm	30, 40, 50	4.56	1500/0.12°	1.4, 1.2, 3/35	10 × 13	512 × 512	4.56 × 4.56 × 4.56 μm	3500(Sr/d)	[27]
SR-μCT	Wood	Microstructure changes under force and stress	DORIS III, DESY (20 × 3)	9	2	720/0.25°	–	1 × 1	1536 × 1024	1 mm^3^	–	[88]
SR-μCT	Wood		TOMCAT, SLS	11	2.15	720/0.25°	0.3/12	3.58 × 1.2	2048 × 2048	1.7 × 1.7 × 1.7 μm	0.7 (Sr/d)	[32]
SR-μCT	Wood	Microstructural decay due to fungi	TOMCAT, Swiss	15	20	2001	0.13/4.2	1650 × 1650 μm	1560 × 2160	0.65 × 0.65 × 0.6 μm	0.5 (Sl/d)	[31]
SRP-μCT	Wheat	Host Interactions	BMIT-CLS, SK	20		1800	0.8/24	1 × 1		13.12 μm^3^	20 (Sl/d)	[53]
SR-μCT	Tomato leaves	3D laminography	ID19 of ESRF, France (60 × 15)	18	0.75	1200	0.5/10	1.54 × 1.54	2048 × 2048	750 × 750 × 750 nm	35 (Sl/d)	[86]
		CO_2_ gas exchange in leaf		20.5	0.75	–	–	150 × 150	–	1.4 × 1.4 × 1.4 μm	3, 4.5, 10.5, 21.5 (Sl/d)	[89]
SR-μCT	coast redwood samplings	Embolism In the xylem	ALS, Berkeley	15	4.5	720/0.25°	12 min	5 × 5	4006 × 2672	175 × 175 × 500 μm	–	[23]
SR-μCT	Ggrapevine	Xylem vessel refilling		24	0.65	1025	60 min	1.7 × 1.7	–	0.65 μm3	–	[107]
SR-μCT	Sunflower	Drought-induced embolism in stems	SYRMEP Trieste (120 × 4)	22	2.0	2048/1°	250 ms/6 min	–	–	–	10 (sl/d)	[20]
SR-μCT	Ggrapevine	Visualizations of Drought-Induced Embolism	ALS, Berkeley (251 × 8) µm	15	4.5	720/0.25°	25 min	–	4006 × 2672	–	–	[21]
SRP-μCT	Submerged leaves	visualise gas films on submerged leaves of common cordgrass	TOMCAT	12	0.375	2001	10 min		2048 × 2048	0.375 × 0.375 × 0.375 μm	1.4–4.0(sl/d)	[25]
SR-PCI	Woody herbaceous plant leaves	With iodine contrast agent	APS,IL USA 207 × 15 μm	7–20	1.66	–	0.4/-	5 × 5	2048 × 2048	–	5500 (Sr/d) 1–10 (Sl/d)	[108]
				10–60	3.56	–	1.7/-		1392 × 1040	–	5500 (Sr/d) 1–20 (Sl/d)	
Seeds												
SR-inline phase TM	Maize	Internal changes in feature	ID19, ESRF (60 × 15) μm	17.6	5	1000	50 s/20 min	–	2048 × 2048	5 × 5 × 5 μm	100 (Sl/d)	[26]
SR-XTM	Arabidopsis	Intercellular void network		21	0.3	800/0.225°	2 s/26 min	0.6 × 0.6	–	5 × 5 × 5 μm	14,500(Sr/d)and 1.3, 3.3, 6.3, 10.3(Sl/d)	[2]
HXRTM	Fossils & modern horse gram	Seed coat thinning with time	I132 of DLS UK (14.5 × 19) μm	15	–	4000	0.15, 0.2, 0.25 s/16 min	–	5120 × 5120	1 × 1 × 1 mm	0.5	[30]
SRP-μCT	Rapeseeds	Distribution of storage oils	ESRF, Grenoble (200 × 200) µm	19	0.75	1200	0.1 s/2 min	–	–	0.74 μm^3^	9 (Sl/d)	[86]
SR-XTM	Fossil seeds	Internal features of fossil flowers	TOMCAT, Swiss	10, 12	0.37, 0.65, 0.74	570–660	–	0.85 × 30.7	10 × 20 μm	0.65 × 0.65 × 0.65 μm	–	[84]
SRP-μCT	Fossil flowers	Cretaceous fossil inflorescence	BM05 ESRF	20	0.75	4000	1.2 s/80 min		FReLoN 8.8 μm		2(Sl/d)	[75]
SRP-nCT	Fossil flowers	Cretaceous fossil inflorescence	ID22-NI ESRF	29.5	0.76	2000	0.8 s		FReLoN 20 μm		4.7, 4.8, 5.2, 6.2 (Sl/d)	[75]
Soil and roots												
SRP-μCT	Barley root hair in soil	Importance of root hairs on pore structure development at the root-soil interface	I13 DLS UK (1.7 × 1.7)	15 to 21	1.6	1601	0.15 s/ 4 min	4 9 × 3.5	2560 × 2560	2 × 2 × 1 mm	6.35(Sl/d)	[41]
SR-μCT	soil aggregation	Soil aggregation in an Ultisol	BL13W1, SSRF, Shanghai (50 × 5)	28	9	430	–	2.70 × 3.2	1052 × 1052	9 × 9 × 9 μm	–	[34]
SR-μCT	Ultisol under Wetting and drying	Intra-aggregate microstructure		24	3.7	1300/0.10°	1.8/39	–	1700 × 1700	541 × 541 × 541	–	[35]
SR-μCT	Quantification of aggregate	Effect of vegetation on strcture		24	3.25	550	30 s		2048 × 2048	3.25 × 3.25 × 3.25	12 (sr/sl)	[95]
SR-μCT	Two soil types of states USA	Characterization of soil microaggregates	ALS, Berkeley (251 µm × 8 µm)	11	20	1800	1/30	–	–	325 nm	0.8 (Sl/d)	[38]
SR-μCT	Soil	Flow of sand	APS, 6.0 (50 × 5)	33.70	17.1	720/0.25° two pass	1.4/16	11.1 × 3.6	1317 × 1335	650 × 650 × 211 mm	–	[42, 43]
			APS 1.5 (1.5 × 1) and (200 × 40) µm	33.69	6.7		5/60	4.39 × 3.45	1300 × 1330	658 × 658 × 517 mm	–	
SR-μCT (KI contras)	Sand	Water content on compaction	BL13W1 SSRF	20	0.65	1200	–/150	13 × 13	2048 × 2048	0.65 × 0.65 × 0.65 μm	–	[36]
SR-μCT at K-edge	Soil	Locate organic matter	HARWI II, Germany (50 × 10)	30, 70, 78	9.77	–	–	–	4.89 μm	3.8 × 3.5 × 3.30 mm	–	[109]
SR-μCT	Two soil types	3D pore network of grassland and tilled soil	DESY, Germany	Tilled: 21.5, grassland: 24	3.2, 5.4	0.5°	–	–	1536 × 1024	400 × 400 × 400	–	[76]
SR-XTM	Soil-root	Growth of wheat root hairs	TOMCAT	20	1.0	1501		–		500 × 500 × 500	–	[24]
Food												
HRXTM	Soft cereal foods	Impact of protein reinforcement on the deformation	BM05, ESRF, Grenoble	19	11 × 11	–	2 s	11 × 22 5 × 10 1 × 2	2016 × 2016	11 μm 5.5 μm 1.1 μm	–	[102]
SR-XTM	Bread	Bubble growth and foam setting		18	–	400	0.02/0.13			628 × 628 × 256 mm		[44]
SR-XTM	Pome Fruits (apple, pear)	Gas network architecture	ID15 EPSRF (3.2 × 3.2)	25	2.5	900	1/0.5		2048 × 2048	1.9 μm^3^	3800(Sr/d)	[110]
SR-PCI		Characterization of fruit tissue	ID19, EPSRF	18	–	700	–	0.7 × 0.5x 1 mm^3^	-	0.95 μm^3^	20.7	[74]
SR-μCT, SRP-μCT		Gas exchange pathways		18	1.4, 5.1 PCI: 0.7	1200	0.5/10	1.43 × 1.43	2048 × 2048	712 nm	10,000 (Sr/d), 3.5 (Sl/d)	[86]
SR-μCT	Extruded cereal & biscuit	Internal structure		17.6	6.5, 7.5, 16.2, 25.8	2000–5000	0.2/15	–	2048 × 2048	7.5 μm (2048 × 2048 × 1024)	–	[46]
SR-μCT	Noodle dough	Characterization of bubbles	BMIT CLS	25	8.75	600/0.3^0^	0.04/1.10	–	4000 × 248 pixels	10–97 voxel	80 (Sl/d)	[101]
SR-μCT	Ice cream	Temp dependence microstructure	(I13-2) DLS, U.K	15 to 30	0.8	900	0.1/1.5	–	2560 × 2160 pixel	2 k × 2 k × 2 k	3.5 (Sl/d)	[45]
SRP-μCT				15 to 30		3601	0.1/6	–				[105]
SR-μCT	Wheat flour	Bubble size distribution in dough	BMIT-BM 05B1-1 CLS, SK 40 × 5 mm	18	8.75	350/1.5°	0.2/2	–	530 × 2530	7–25 voxels	147 (Sl/d) 2620 (Sr/d)	[77]
SR-μCT	Coffee Beans	Voids/pore volume distribution of green and roasted coffee beans	SYRMEP, Trieste (Italy)	19, 20	4.5	1440/0.125^0^	–	18 × 12	4008 × 2672	1 mm^3^	20 (Sl/d)	[47]
SR-μCT	Chocolate	Migration pathways through cracks and voids	(DESY) Hamburg	13	1.0	1800	1/20	1.8 × 1.8 mm	3056 × 3056	100 μm	–	[48]
SRP-μCT	Fish	Histology	APS, ANL USA	13.8, 16.2	1.43	2048	20/20	–	–	0.743 μm^3^	3 (Sl/d)	[103]

*Sl/d* sample to detector, *Sr/d* source to detector distance, *μCT* micro computed tomography, *TM* tomography

**Fig. 5:**
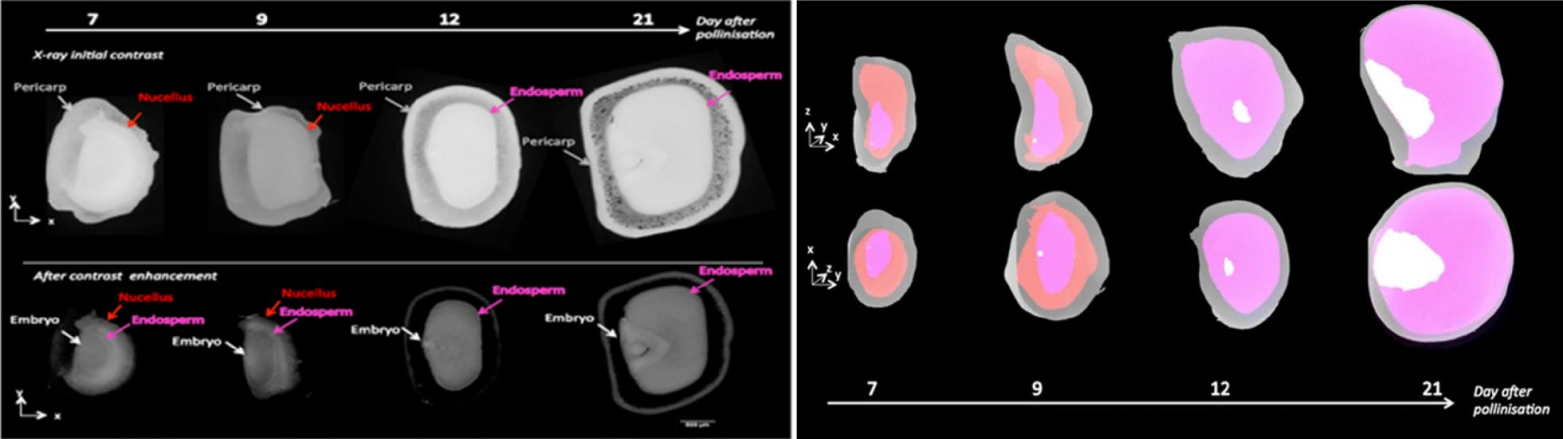
Left: X-ray images and Right: 3D segmentation of maize seeds at four different developmental stages corresponding to 7, 9, 12 and 21 days after pollination [26]

Earlier, conventional histology only was used to study the developmental stages of crop seeds which is a skilful and time-consuming job, but Rousseau et al. [26] team had imaged for the first time the development stages of maize (Table 1) seeds after days of pollination (DAP—7, 9, 12 and 21). The results were compared with conventional histology and demonstrated a good match for the estimation of the length of different components of the seed. The simple thresholding was used to quantitatively segment maize seed into four components of the seed (embryo, endosperm, nucellus, and pericarp) from 7 to 21 DAP (Fig. 5). The lack of cellular resolution as compared to conventional histology can be overcome by higher spatial resolution by imaging a small part of the sample in a local tomography mode inside the same type of seeds [26]. Use of statistical tool of fisher ratio as a contrast index in their experiment enhanced the quality of the images and segmentation strategy.

**Fig. 6:**
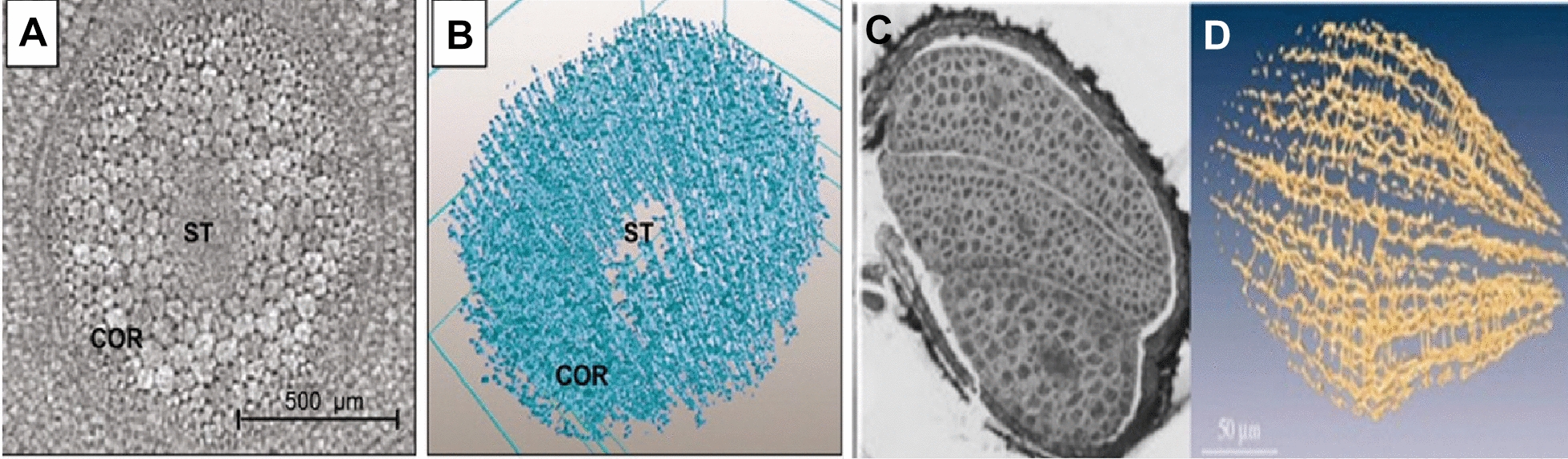
3D model of void spaces (**A**, **B**) in the hypocotyl of a developing rape seed (*Brassica napus*) [86] Copyright (2006) National Academy of Sciences and Virtual slice (**C**, **D**) of Arabidopsis seeds, void network and reconstructed voids in phase contrast imaging [2]

A seed is a living entity, which means it respires even after harvest or in storage and can retain its germination. Gases like oxygen are required for support of life and trapped gases inside seed structure or components might play important role in germination. Therefore, Cloetens et al. [2] had used synchrotron (Table 1) X-ray imaging of Arabidopsis seeds and concluded that intercellular void network provides a transport system for easy gas exchange in embryos and storage space for the oxygen. Produced results were a 3D representation of the local electron density which is directly proportional to the refractive index of material for hard X-rays [2]. The investigation also claims that stored oxygen plays an important role in the onset of germination, during imbibition (Fig. 6). Dhondt et al. [85] had also scanned different *Arabidopsis* plants until 13 day after sowing at a spatial resolution of 10.4 μm like Cloetens et al. study [2], (Table 1) and revealed many detailed morphological features such as furrows in the hypocotyls, branching of trichomes, the pollen sacs on the anthers, and the stigma on the flower style. Seeds like rapeseed are small and may appear homogeneous at the macroscopic scale, but their growth and physiology are dependent on several microscopic features, including cell shape, cellular water potential and the presence of intercellular voids [86]. SR-µCT has provided evidence for the existence of voids (Fig. 6) and its spatial and geometric complexity has a strong influence on endogenous gas transport and flux, and so may affect the local respiratory activity and seed growth [87]. Seed structural characteristics may responsible for gas exchange and it can be occur through corner cavities located in dry seeds [2].

### Plants

The living plants have unique internal features to transport water and nutrient from roots to a body of plant (leaves and fruit). The complex water and gas transport phenomena that occur in plant tissues were studied in detail using SR by mapping porosity, microstructure arrangement and the connectivity of the vascular systems. The cellular level information could be gathered easily without any need for additional preparations of samples in plants for analysis [2, 29, 39].

#### Structural, composition and dynamic process

Synchrotron absorption and phase contrast imaging have been used extensively to study structure–function relationship and dynamic processes in plants [1, 14, 27, 39], vessel networks [11, 31, 51, 86, 88], diffusion of gases (CO_2_) in leaves [25, 85, 89], vascular function in live plants (sunflower, oak, redwood, walnut, grapevines and maple) [39], occurrence of xylem cavitation in rice and bamboo [17], and stress physiology [90].

**Fig. 7:**
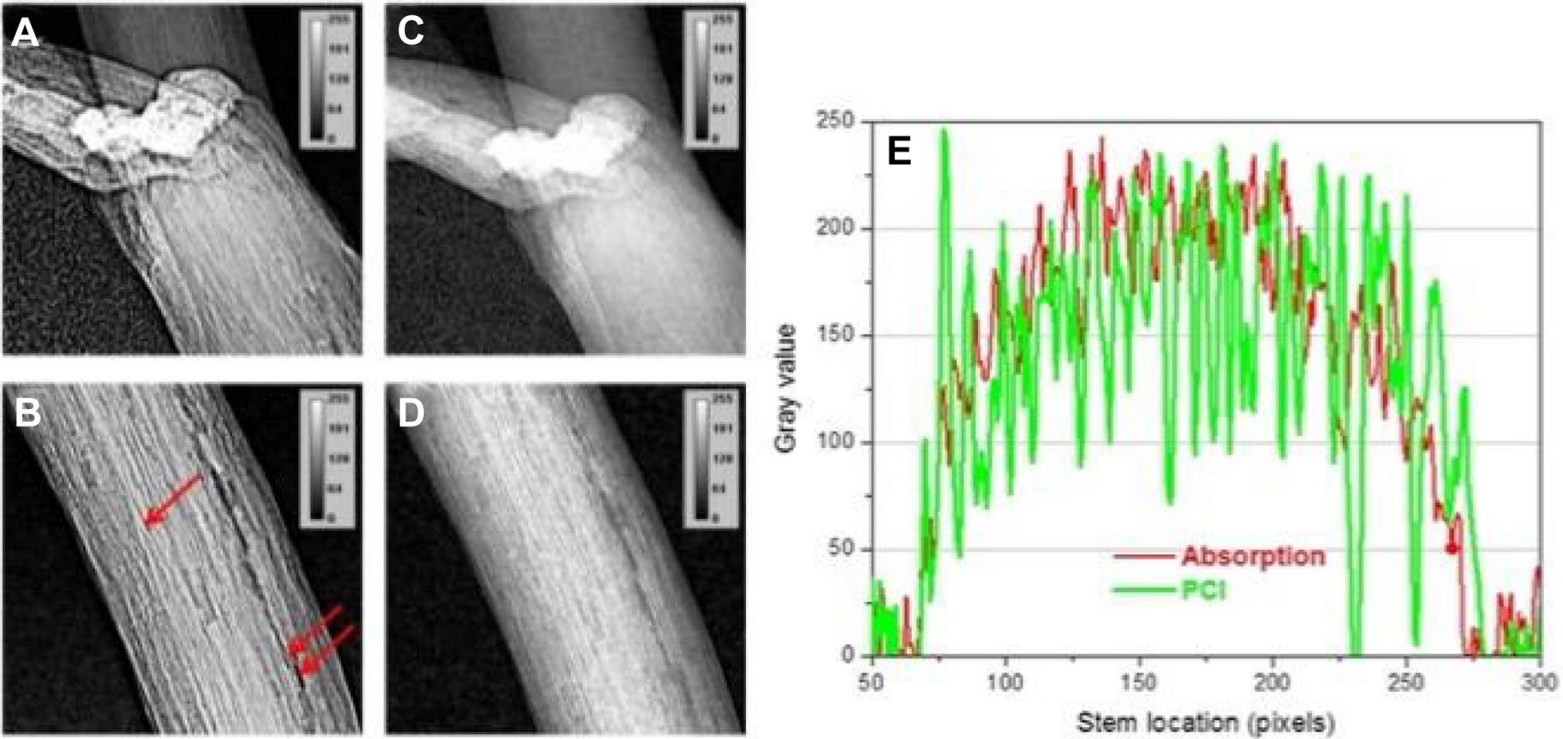
The 3D images showing internal details of canola plant in SRPµCT (**A**, **B**) of plants and SR-µCT (**C**, **D**). Histogram of gray values for absorption and phase contrast are given in **E** [1]

**Fig. 8:**
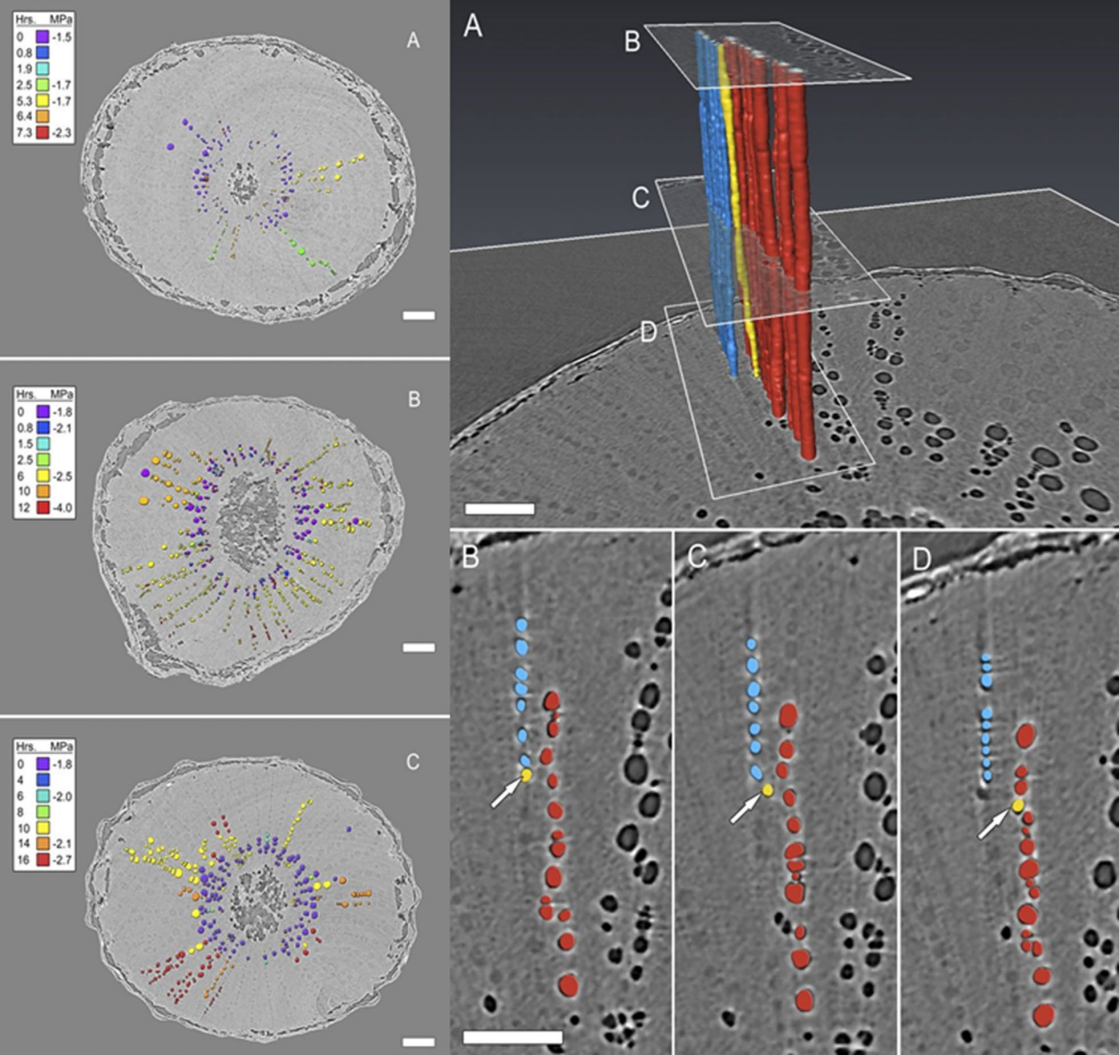
Top: Transverse high resolution CT micrographs of grapevine stems showing the radial, sectored spread of embolisms during the simulated drought experiments. Vessel lumen colour denotes the time point (hours) at which gas was first observed inside the vessel. Bars = 1 mm. Bottom: volume renderings of grapevine vessels showing a pathway for embolism spread between sectors and (**B**–**D**) Vessels in the red sector embolized first and then spread through a vessel (yellow) that drifted tangentially from the red sector to the blue sector [23]

The differential X-ray attenuation is responsible for visualizing the difference between water, and air, and this property makes synchrotron X-rays perfect to study how water refills xylem vessels in plants [91]. As mentioned earlier phase contrast imaging is best suited for soft tissues because it has lower absorption for X-rays, therefore it was compared with SR-μCT (Table 1) by one group at a Canadian light source and revealed microstructural details of the canola stem such as cavitation and surrounding tissues of the vessels [1]. Phase contrast imaging was found best to generate better contrast in images to reveal finer details (Fig. 7) and the xylem water refilling process. Whole canola plants were scanned during this experiment, so X-ray energy optimization was carried out and fixed at one energy 18 keV for above ground portion, and two energies 24 keV (clay soil medium) and 38 keV (sandy clay loam medium) below ground portion [1]. A group from Shanghai also carried out a similar study and found (Table 1) that water refilling process is not the same throughout all parts, because cavitation occurs to different degrees under different dehydration conditions [17]. Similar kinds of studies were reported for study of drought-induced embolism in stems (Table 1) of grapevines [21, 33, 92], sunflower [20], wood microstructure [88], and coast redwood [23]. The purpose of these studies was to quantify resistance to embolism in plants, which is important for understanding their drought response [33] and whether plants can rapidly refill or not, in embolized conduits during recovery from drought stress, which is an important component of their survival in a rapidly changing climate [23]. Embolism affects long-distance water transport through plant xylem which often coincides with drought. Researchers were able to study the dynamics of drought-induced embolism in plants (grapevines) and produced three-dimensional, high-resolution, time-lapse observations of embolism spread (Fig. 8). High-resolution time-lapse SR-µCT images of embolism removal in plant have improved understanding of the final stages of a biophysical process that plays a critical role in drought recovery of plants [23]. X-ray induced damage of about 25% was also reported by one of the groups during SR-µCT scanning of sunflower plants [20]. Choat et al. [23] examined spatial patterns of xylem embolism in saplings of a conifer species coast redwood during cycles of drought and rewatering. They did not find any evidence for embolism refilling in tracheids after drought up to few weeks in their study on basis of SR-CT data and provided a reason that, conifer species lack an active refilling mechanism. Also, another reason is that their xylem lacks the specialization in cell function necessary for such a process, which can be observed in angiosperms. Based on these salient findings, several experiments could be run at synchrotrons, ranging from the characterization of drought resistance of different crop species to the evaluation of different treatments on species hydraulics.

The novelty of synchrotron imaging was proven when it was used (Table 1) for the first time for the characterization of gas film retained on superhydrophobic leaves of submerged plants, which revealed how wetland species can survive in submerged conditions [25] and such kind of studies have opened the new area for its application. The generated data could be used for study of structural changes in the cuticle that occur with a time of submergence and then how these changes affect hydrophobicity and the capacity to retain a gas film under water [25].

Manufacturing good structural and utility-based wood is important for any industry, therefore being a natural resource, the study of its structure is of prime importance. The synchrotron has been used in a study of wood structural components, environment stress, and fungal decay at the micro level [31, 32, 88]. The detection of boundary surfaces between neighbouring cells and interconnectivity in wood specimens was challenging with conventional scanning electron microscopy (SEM) and light microscopy [70], but SR-μCT made it possible (Table 1) to map those components. The acquired data were used in the estimation of volumes of water transported in vessel cells and visualized vessel networks in wood samples to understand the effects of wounding and environmental stresses on the xylem structure and water transport system in wood. Forsberg et al. [32] used a novel application combining SR-μCT and digital volume correlation for the deformation analysis of wood under bending stress (Table 1) and developed a methodology for study of fresh plant tissue fracture in structural timber. This was one of the first attempts to use synchrotron to study the influence of anatomical features on the fracture behavior of wood and quantification of deformations during in situ micromechanical experiments.

The high-resolution imaging data generated by synchrotron imaging can be used in validation of developed models like light propagation, CO_2_ diffusion, and photosynthesis simulation, therefore, Ho et al. [89] and Verboven et al. [87] imaged tomato leaves (Table 1) to visualize micro leaf structure for gas exchange and related them to leaf optical properties for validation of developed models. Radiation dose in synchrotron imaging is dependent on photon energy, attenuation coefficient, and density of a material, therefore soft tissues are susceptible to damage by X-rays [27]. Therefore, to minimize the radiation doses, Matsushima et al. [27] used three photon energies 30, 40, and 50 keV in scans of three varieties of rose peduncles. In the selected photon energy ranges, linear absorption was negligible for soft plant tissues. Both SR-μCT and SRP-μCT methods (Table 1) were used in this experiment. In SRP-μCT due to edge enhancement, the cell walls of each single cell were distinctly depicted and clear discrimination was achieved between fully intact cells containing cytoplasm and water-filled cells and air filled objects [27]. The tissue damage was observed for one variety due to more exposure time at 30 keV and 40 keV energies as compared to 50 keV, where effective surface dose was less. It was clarified from the results that the involvement of distinct structural differences in a vascular bundle and typical peduncle pith structure was responsible for the evaluation of different vase life for the selected rose varieties.

#### Infection and disease detection

**Fig. 9:**
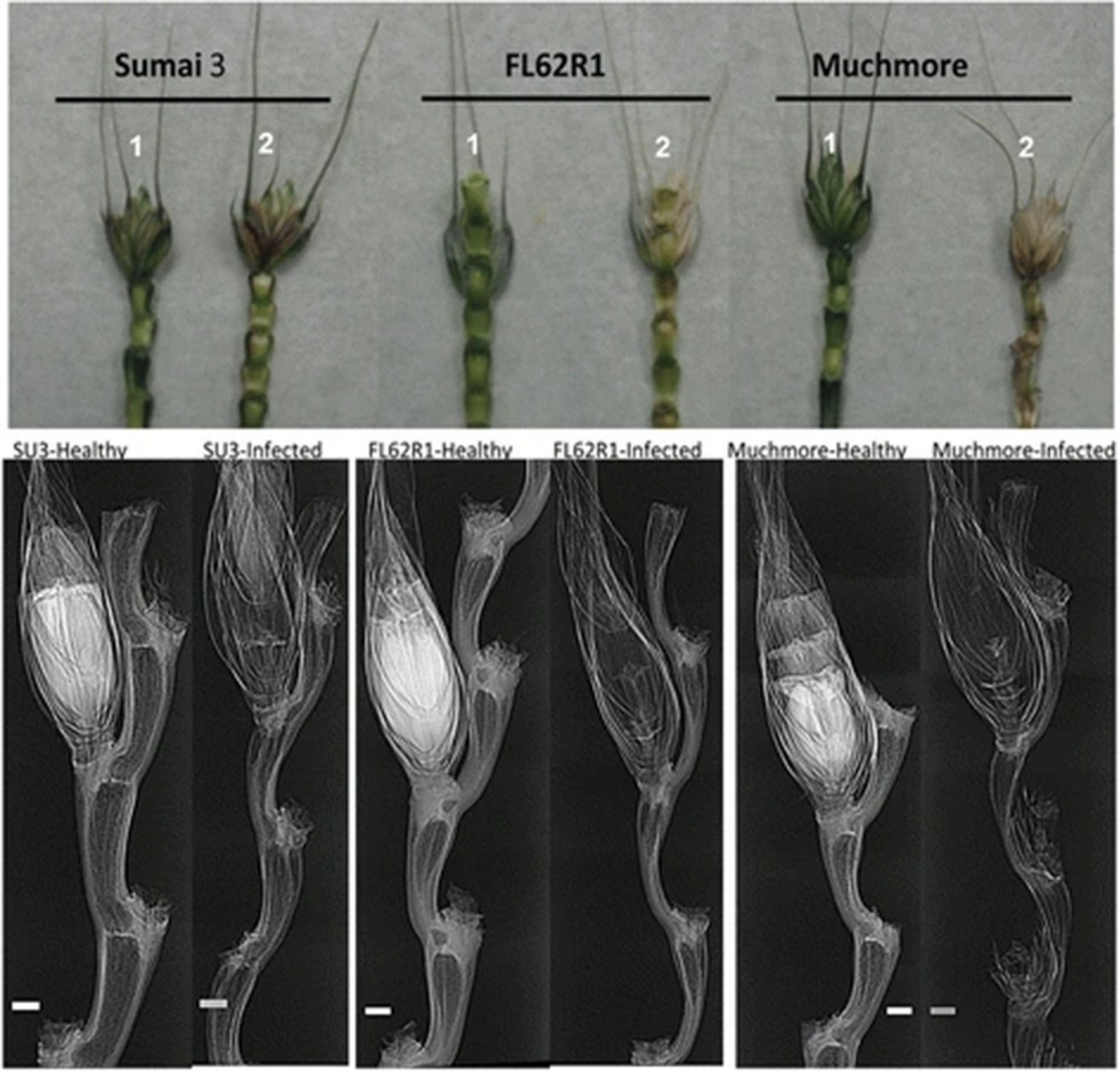
Phase contrast X-ray images of healthy (1) and infected rachis (2) of wheat cultivars at four days after inoculation with FHB [90]

Capabilities of synchrotron imaging were assessed for disease and infection detection in live plants in a non-invasive way, like visualizing changes in internal features of plants, for detection of fungal infection or decay in the wood [31], fusarium resistance characterization in wheat [51, 53], and understanding insect anatomy for control management practices [50]. Fusarium is one of the five top priority diseases of wheat in Canada, which not only reduces yield but destroys protein content and results in toxin accumulation in grain [53]. Synchrotron imaging was found useful in studying plant-pathogen interactions, especially in determining the role of cell wall structural characteristics in response to fungal infection [51]. Therefore, Lahlali et al. [50] mapped structural differences in spikelets of wheat, because it plays a significant role in resistance to Fusarium. Differences in mass densities and phase contrast signals between healthy and infected spikelets are clearly visible (Fig. 9). In one cultivar (Muchmore) structure of rachis was altered and became more transparent to synchrotron X-rays which was a sign of infection. Wheat typically has resistance to pathogens, based on which wheat is classified into five types (Type‐I to Type‐V). High-resolution X-ray images can be used in the characterization of wheat into five categories on basis of resistance. Brar et al. [53] scanned seven genotypes of wheat using SRP-μCT (Table 1) for detection of changes in voids, volume fraction, and voxel intensity due to infection of *Fusarium graminearum*. Their results revealed that rachilla and rachis nodes together provide significant resistance to pathogen spread because inoculated spikes had higher void space fraction and lower X‐ray attenuation compared with controls. They also stated that a pathogen can still invade internodes, but this structural reinforcement significantly impedes disease progress. The disintegration of host tissue was observed due to infection and failure of the ovary to develop in the infected floret were observed in the images. They had also developed Python-based scripts and produced histograms with voxel intensity for each ROI (voxels ranging from 99 to 145 million). They found that one genotype (*CDC Alsask*) which was more susceptible to FHB *(Fusarium graminearum)* had higher void space volume among seven genotypes. As discussed earlier, structural integrity is important in wood used in any industry, therefore it is usually treated for better resistance to decay and more service life. Changes to wood structure at micron lever under fungal decay is of prime importance. Hence, synchrotrons have been found useful in mapping changes in the wood structure under fungal infection. Gilani et al. [31] used SR-μCT (Table 1) for wood at the micro-scale level and evaluated alterations in density distribution after incubation of samples with two white-rot fungi. They were able to develop an algorithm for quantitative study of the density changes in the wood cell walls after different stages of fungal decay and mapped internal changes to establish relationships to describe variations in porosity and density.

### Soil medium and roots

Good soil structure is essential for supporting plant life to provide essential nutrients and ensure sustainable agriculture. The quantification of pore network, microstructure properties and their relationships are required to enhance our ability to predict changes in soil ecosystems [93]. The hard X-rays of synchrotron have been used to investigate and characterize pore networks, arrangement of particles, the effect of organic and inorganic matter, root hairs, and soil state under alternate dry–wet conditions [24, 34, 38, 42, 43, 71, 76, 94–96]. All the experiments reviewed in soil application were carried out at higher X-ray energies up to 78 keV and high resolutions (0.65,1.0 μm) in comparison to experiments with plants, fruits, and foods [24, 36, 76].

#### Internal structures of soil aggregates

Study of internal arrangement of soil aggregates is important because soil structure provides pathways for the transport of water, nutrients, gases, and habitats, therefore, it is a fundamental property of soil fertility and quality [97]. The synchrotron absorption imaging and computed tomography were used to investigate the arrangement of soil aggregates and changes in soil pore network due to fertilizer application [95], porosity due to dynamic change in water content [94], and effects of type of soil on water flow and behavior under physical force on structure [38, 76]. Soil erosion is a major problem across the globe, where native grassland is converted as farmland for growing food crops. In recent years, a lot of efforts were made to improve the health of these eroded land by growing natural vegetation. In the semi-arid region of China similar efforts were made for stabilizing abandoned croplands with natural vegetation for different lengths of time. One of the aims of a study was to investigate whether natural revegetation on abandoned cropland could improve the stability of soil aggregates, and whether their microstructure could become more connected during natural revegetation. Zhou et al. [34] used SR-CT for quantification of complex aggregate microstructure in soil and its relationship to vegetative restoration. It was found that aggregate microstructure was modified substantially during the vegetative succession and the porosity of the aggregates increased from 9.2 to 45.9% for samples of selected cropland sites. In this study, pore sizes and shapes were expressed based on equivalent diameters and shape factor (F = 0.2–0.5) and classified into four size classes (< 30 μm, 30–75 μm, 75–100 μm, and > 100 μm) and three shapes (regular, irregular, elongated). It was concluded from the results that aggregate microstructures were more at abandoned cropland sites than at active cropland sites.

**Fig. 10:**
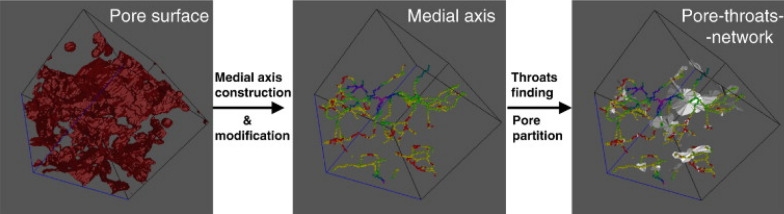
Schematic sequential diagram of the pore–throat network construction [34]

Soil micro aggregates microstructure plays an essential role in chemical heterogeneities, microorganism distributions, origin and development of hot spots in soils [38]. Hence quantification of soil aggregates has become of prime importance. The analysis protocol for the morphometric characterization of complete soil micro aggregates had been developed (Table 1) for two distinct soil types of regions; one from Kansas, primarily composed of inorganic particles, and one from Barrow (Alaska) dominated by plant fragments (Fig. 10). In the study they found that the use of local thickness analysis, commonly used in medical imaging for porous material characterization was good approach. Local thickness can be defined for a voxel as mean diameter of the maximum inscribed sphere in the structure that contains the voxel [38]. The Kansas sample had shown typical interstitial pore space, created by the aggregation of rounded mineral particles and aggregates whereas a more complex microstructure was found in the Barrow sample with a strong organic component which was verified by variation in attenuation values, due to the high percentage of plant fragments. The Morphometric analysis revealed that Barrow aggregate had high porosity (81%) as compared to Kansas aggregate (43%) which typically has granular composites.

**Fig. 11:**
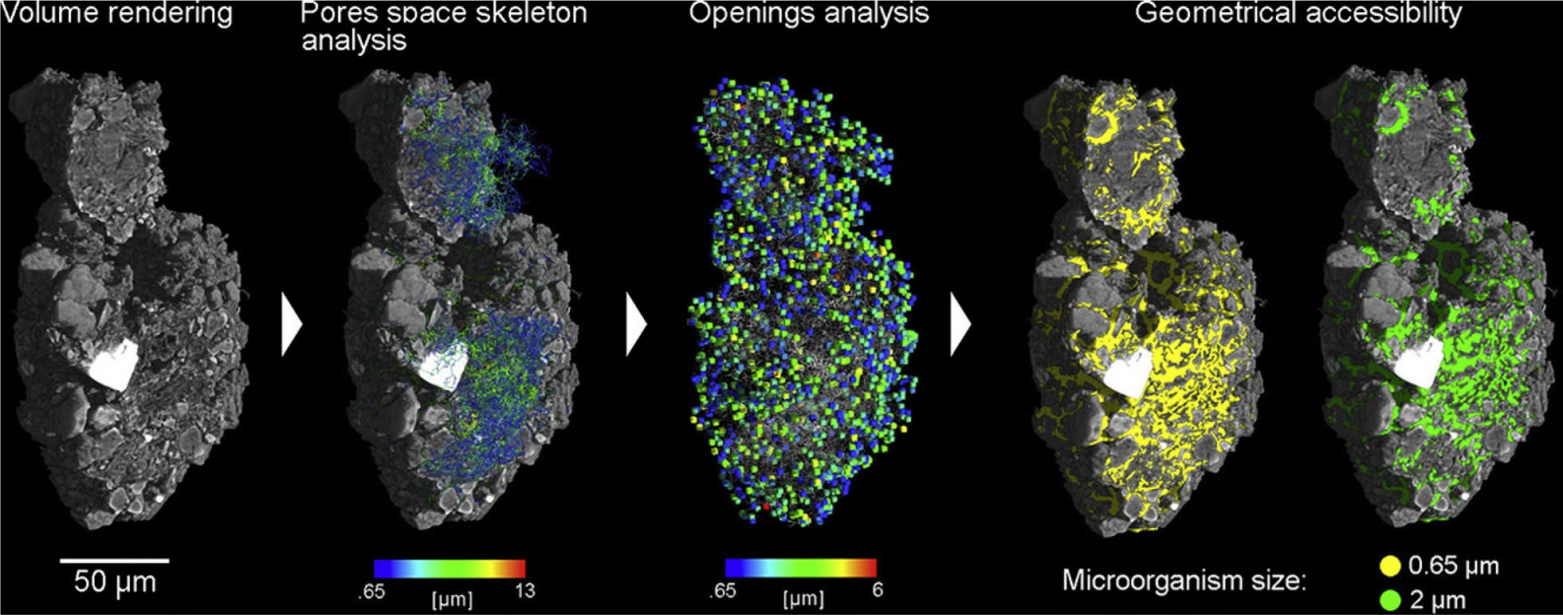
Steps involved in image processing of soil aggregate from left; volume rendering, pore space analysis, opening analysis and geometrical accessibility [38]

The long-term use of fertilization practices can improve soil aggregation through associated increases in organic matter over time, but organic and inorganic fertilization have different influences on aggregate structures [34]. The aggregate stability under organic and inorganic fertilizer application can be studied using synchrotron [93]. They did analyzed soil samples treated with NPK (chemical fertilizer) + OM (organic matter) and NPK and CK (no fertilizer) (Table 1) at Shanghai synchrotron facility. They had used open-source platform Imagej for reconstruction with a polar transformer plugin and a Matlab program for ring artifacts removal by masking the Fourier transformed image. Use of algorithms like morphological erosion based on the algorithm (LKC algorithm) was found in their study for pore–throat-network construction (Fig. 11). Measurement of porosity, nodal pore-size distribution (PSD), pore throat size distribution (TSD), effective throat/pore radii ratio, path length distribution, and tortuosity (ratio between the path length and the straight distance between the ends of the path) were also determined by developed methodology of image processing. Pores were classified into macropores (> 500 μm) and mesopores (≤ 500 μm) according to their equivalent diameters in their study [34]. The results of image analysis revealed that microstructural pore properties were almost the same for NPK and CK treatments. The number of pores, number of pore throats, and number of paths between adjacent nodal pores were all significantly decreased by the NPK + OM treatment relative to the NPK and CK treatments. X-ray attenuation coefficients of organic matter fall between water and air, hence the mineral matrix, making phase separation problematic [76]. Therefore, Peth et al. [76] used monochromatic SR- μCT at photon energies above the absorption edge (78 keV) and photon energy below the absorption edge (70 keV) to locate soil organic matter in the stained soil aggregate samples in relation to soil structure, where attenuation contrast is optimal for distinguishing other soil constituents.

As discussed earlier, vegetation has significant impact over soil aggregate structure, similarly wetting and drying of soils is also responsible for changes in aggregation and soil structure [35], hence there is good scope to enhance understanding of changes in topsoil microstructure due to above phenomena. The soil aggregates of 3–5 mm were scanned with an SR-μCT at the same beamline (Table 1) and revealed that wet aggregate stability and tensile strength were closely associated with the pore characteristics. The pore characteristics (pore space larger than 75 μm) and soil clay content accounted for as much as 99% of the variation in both wet aggregate stability and tensile strength [35]. The pore-space geometry of soil aggregates determines the transport of water, gases, and nutrients [76], therefore, 3D visualization and quantification (Table 1) were carried out on two distinct soil samples which went through different management practices (tilled and grassland). In the study, sample structure was quantified on basis of pore-size distributions (PSDs), throat-area distributions, effective throat, frequency distributions of pore channel lengths, widths, and flow path tortuosity. It was determined that the soil aggregate of the tilled site showed more gas and water transport limiting micromorphological features compared to grassland management system. In continuation to this, even agriculture management operations like tillage have an influence on the microstructure of soil aggregate. The study of such mechanical forces has become necessary, therefore, conditions like compaction were been simulated on beads, natural sand, and clay and data acquisition on them was carried out [36]. Two photon energies were used 18 and 20 keV during image acquisition for quick determination of the water distribution in the selected material. Quantification of water (10.2 and 9.3%, for glass beads and sand, respectively) from image segmentation at 0.65 μm/pixel was obtained which was comparable with those measured by the oven-drying method (9.7 and 9.4% for the glass beads and sand, respectively). Contrast agent iodine was used in this study and it was stated that addition of 20% by weight of iodine-based contrast medium can increase the greyscale value of the liquid and enhance the air–water contrast which contributes to (semi) automatic segmentation of water phase.

#### Soil–root interactions and dynamic process

**Fig. 12:**
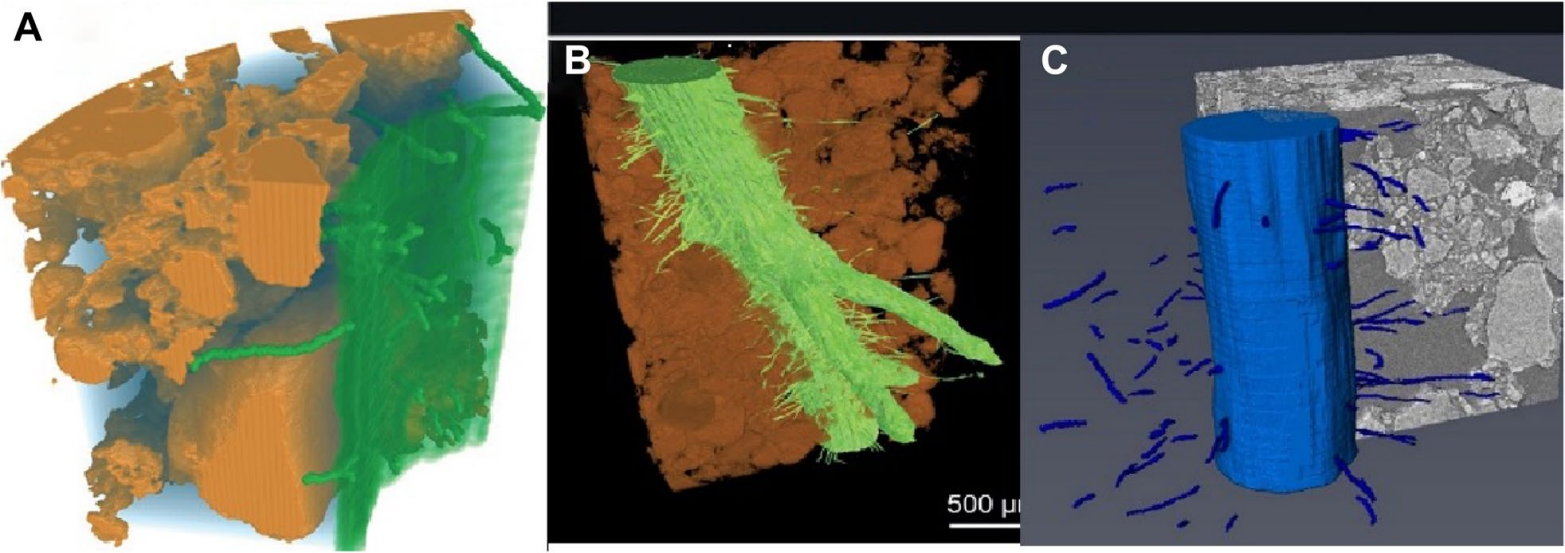
**A** 3D volume mesh is generated, with root hairs, soil, root surface and water, **B** section of a seminal root including lateral roots and root hairs and **C** 3D rendered barley root and hairs showing the surrounding soil [24, 41]

The soil ecosystem contains microbes, roots, nutrients, and water that supports plant life, therefore, synchrotron X-rays were used to study soil root interaction, dynamic uptake process, drainage [42], root hair and aggregate orientation [24, 41], and water distribution in compacted soil [36]. The role of root hairs in plant and soil science remains poorly understood, which limits the targeted selection of root hair traits, particularly for enhanced nutrient acquisition in the field [24]. Root hairs play important role in the uptake of sparingly soluble nutrients, especially in nutrient-deficient soils. An earlier study of root hairs has been conducted in artificial hydroponic gel systems, by destructive washing of roots from soil, and a non-destructive approach to this problem was necessary. Therefore, synchrotron X-ray imaging was used by Keyes et al. [24] to uncover the three-dimensional interactions of root hairs (Fig. 12A, B) in soil. They had investigated phosphate uptake by root hairs based on the geometry of hairs and associated soil pores, which was accomplished by the finite element (FE) method using COMSOL(ScanIP). This study has improved the understanding of how root hairs can be modelled at the plant and crop scale, and indicated that previous modelling studies should be re-visited using imaging based (like synchrotron), multi-scale homogenization approach. They recommended that roots and hairs both equally contribute to phosphate uptake. Similarly, image based mathematical modelling approach was used to provide importance of root hairs on pore structure development at the root–soil interface during the early stage of crop establishment [41] and found that root hairs had a significant effect on soil structure (Fig. 12C) formation and influenced porosity and connectivity for the pores (≥ 5 μm) visualised with synchrotron imaging (Table 1).

Reviewed studies have shown how structural features of soil aggregate structure influence functional characteristics. Similarly, drainage property is associated with physical characteristics of soil. Agricultural drainage not only check the water table but also maintain healthy root zone ecosystem around the crops. Fast acquisition of synchrotron images made it possible to visualize dynamic behaviour, pattern and quantitative analysis of flow processes in a sand [42]. In this study image acquisition was carried out at two beamline operation parameters (Table 1) for two conditions (fast and slow drainage). Nearest neighbour analysis was carried out for quantitative analysis on air phase distribution and found differences in drainage pattern. In fast drainage condition air bubble tend to be few and far apart, whereas closely packed smaller bubbles were found in slow flow condition [43]. The drainage occurs through well connected large pores initially than finer pores, and drainage can occur through finer pores only under high air phase pressure near boundaries. It was also found that greater pore structure formation presents away from the root for the plants with root hairs [41].

### Applications in food

Use of synchrotron imaging tools have gained the momentum in area of food science like agriculture science. Conventional X-ray sources have been used extensively in study of food microstructures and product development as reviewed by Schoeman et al. [98]. The access to synchrotron technology is limited, lack of awareness about advantages of the technology and associated cost might be other reasons why synchrotron was not used extensively in the past. But, now due to increasing number of facilities around the globe, researchers have been able to conduct studies for understanding microstructure of foods and its role in improving food quality particularly its texture and taste. Many food products contain a cellular foam structure, such as ice cream, mousses, sponge cake, biscuits and bread, which needs to be characterized in order to determine the relationships between the structure of the product and its mechanical and organoleptic properties [46]. The microstructure of food products is highly correlated to their physical and sensory properties, which are important to evaluate the consumers acceptance of food [99]. Synchrotron experiments were carried out to map changes in structure of food like bread, noodle dough bubbles [44, 46, 77, 100, 101], cracking of chocolate [47, 48], microstructure changes due to gas exchange in product [13, 98, 102], ice cream stability under varying conditions [45, 103, 104], air pathways in pome fruits [74, 86] and in fish [103]. Food science involves many processing or unit operations which may involve addition or removal of heat, application of pressure and mechanical shear. These unit operations used in food processing have significant impact on food product structural properties and quality attributes. Study of these changes using synchrotron imaging are discussed here and summarized in (Table 1).

**Fig. 13:**
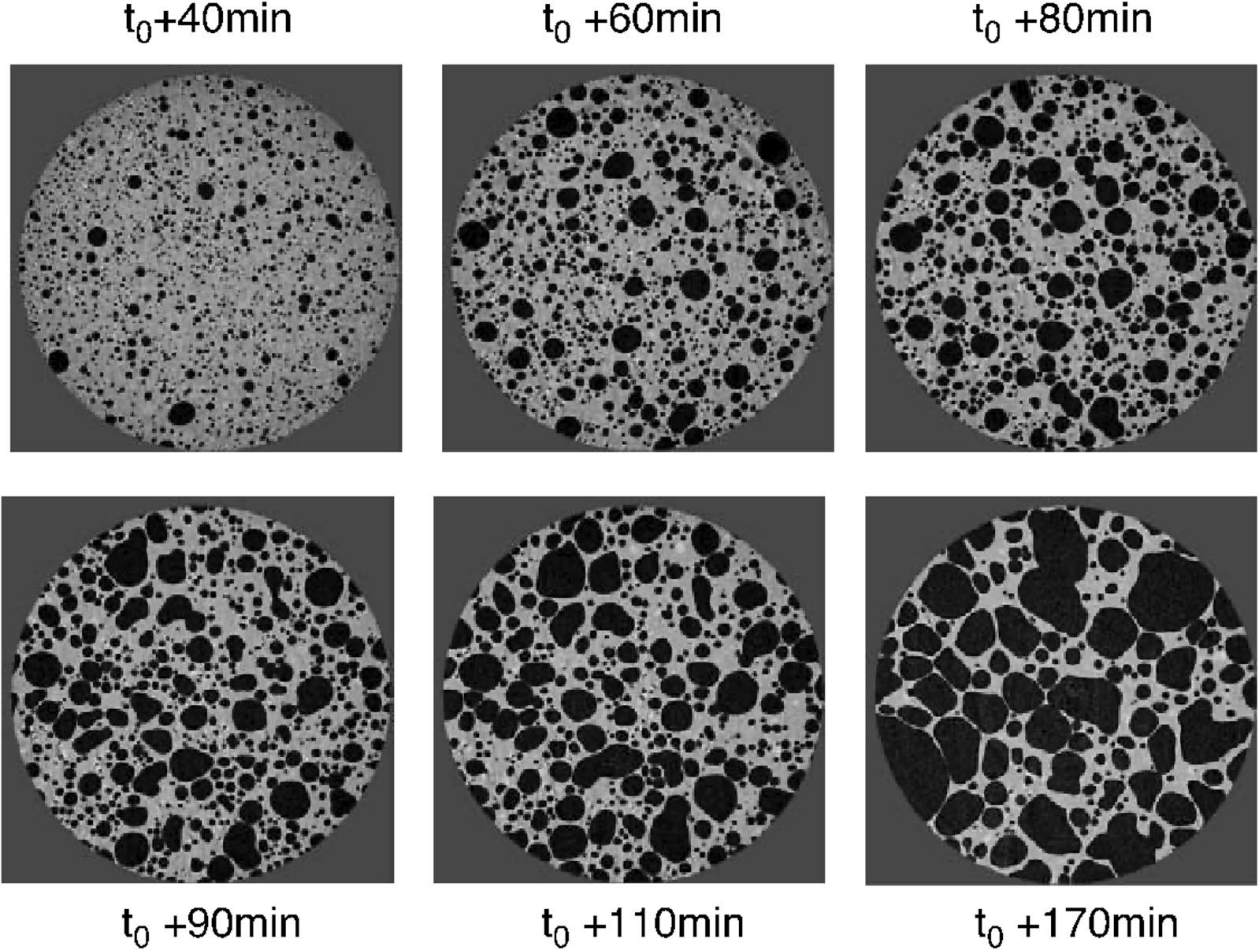
Dynamic bubble growth at increasing time period in dough in reconstructed synchrotron 3D X-ray images slices [44]

Cereal based food products are consumed in the form of bread, noodles and in puffed form across the world which is a staple food to most of the people. Bread is the most common processed product of cereal wheat, and its quality depends on dough quality. Tabletop X-rays have been widely used for studying 3D structure of bread and other stable food products in the past, but these studies have certain limitation and were not able to map rapid bubble formations. The microstructure of dough is challenging because dough is opaque, and the bubbles in dough are extremely fragile and change very rapidly [77]. Study of bubble distribution, size and formation with time is critical, hence has been studied using synchrotron in bread and noodle dough [44, 46, 77, 100, 101]. Babin et al. [44] studied bubble growth and foam setting during breadmaking (Table 1) and revealed that development of gas cell structures during fermentation was critical in breadmaking. The generated 3D image data sets were used successfully in validation of numerical models of bubble growth, and they found clear evidence of bubble formation with time (Fig. 13). The development of gas cell structures during fermentation depends on a critical time initially then coalescence prevails rapidly, and leads to a heterogeneous structure. It was stated that the minor components present in flour may play an essential role in obtaining a desirable bread texture. Their study recommends that synchrotron can be used for the study of rheological properties of doughs and the temperature dependent changes of wheat flour biopolymers which govern the bread baking. Similarly, Koksel et al. [77] investigated bubble size distribution and its evolution in non-yeasted wheat flour doughs (Table 1) and found higher bubble number densities compared to previously reported studies carried out with conventional imaging. Bubble distribution was monitored and measured by customized algorithm developed in MATLAB, and found that distribution had a median bubble radius of 22.1 ± 0.7 μm at 36 min and at the end of mixing around 162 min, the size was increased to 27.3 ± 0.7 μm. This mapped dynamic trend of bubble distribution was indicative of transport of gas in the dough due to disproportionation (a phenomenon by which smaller bubbles disappear and larger bubbles remain or continue to enlarge within the semi-homogenous viscous dough). Results of both studies have contributed significantly to bakery industry in standardization of dough constituents. In continuation to previous studies, Guillermic et al. [101] did rapid characterization of bubbles in noodle dough (Table1). This non-destructive study of bubbles in wheat-flour noodle dough provided a more complete knowledge of the dough sheet's internal structure, and how it originates during processing, and its effect on the overall quality of Asian noodles. Analysis of images revealed that gradient in concentration of bubbles within the dough sheet was present from the core to the sheet edges. Extrusion processed food products or puffed products are fibrous and porous in nature. Extrusion has a significant effect on food product texture, which is an important quality attribute. The cellular structure of a cereal food is an important feature that involves sensory aspects and carries information about the processing and composition of the recipe, therefore, Chevallier et al. [46] studied cellular structure of two products, an extruded breakfast cereal and a short dough biscuit with SR-μCT (Table 1), and compared results with conventional X-ray CT. In this study, image acquisition was carried out at four different resolutions: 6.5, 7.5, 16.2 and 25.8 μm, because their aim was to find out right resolution for determination of quantitative measurements such as densities and thicknesses.

**Fig. 14:**
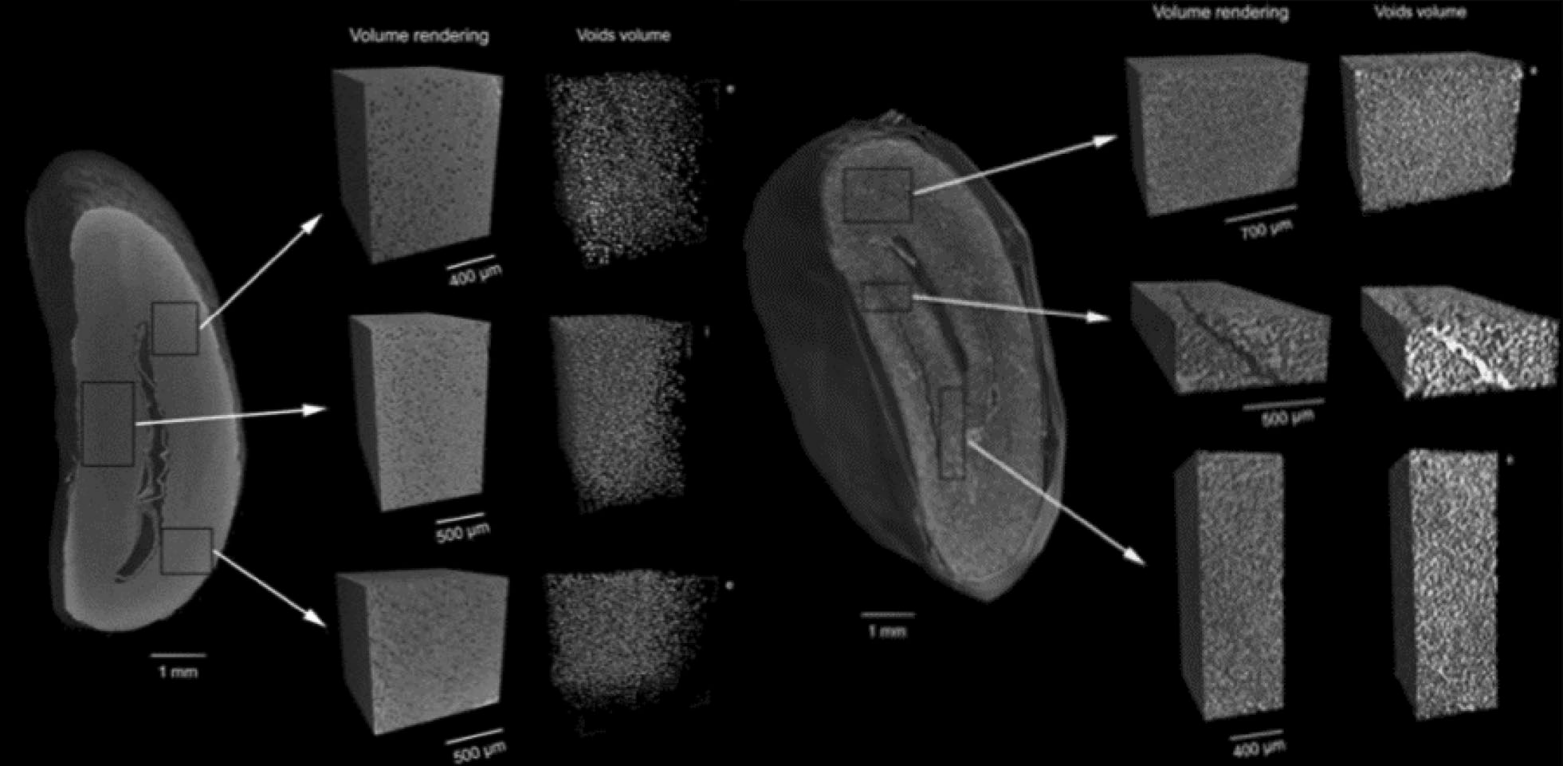
The virtual cut of 3D synchrotron X-ray images, where porosity in non-roasted (left set) is lower than roasted coffee bean (right set) [47]

The SR-μCT produced less noisy images with shorter acquisition time, which is important and compatible with dynamic studies. Both products exhibited quite different cellular structures, Biscuit had lower porosity, with smaller cell sizes, thinner cell walls compared to extrudate snack. The cell density of biscuit was found to be ten times higher than the extrudate snack. Roasting is one of the most common unit operations in food processing, where addition of heat is involved. In coffee processing, roasting is an important step in which green beans are subjected to temperatures up to 250 °C as per desired degree of roasting. The heat-induced reactions have significant effect on changes in the chemical, physical, and structural properties of the raw bean, which, in turn, affect the sensory and texture characteristics of the roasted coffee. Pittia et al. [47] used SR-μCT (Table 1) to investigate morphology and the inner microstructural properties of coffee beans as well as the effects of the roasting on it and found that roasted coffee beans had a higher and more uniformly.

distributed porosity in the order of 40% as compared to green beans 6% (Fig. 14). This type of study has opened the possibility to investigate effect of process parameters on the porosity evolution during roasting and to relate the results with those obtained with other conventional and non-destructive imaging.

Like addition of heat, removal of heat in unit operations such as freezing also alters the structural properties of foods, mostly in dairy products. Textural properties of ice-cream products depend on its structure, but cooling cycles may affect its structure due to crystal formation. Therefore, Guo et al. [45] were used SR-μCT (Table 1) to determine the temperature dependence of ice-cream's microstructural evolution. Results of the study revealed that melting-recrystallization mechanism is responsible for the evolution of ice crystal size and morphology during thermal cycling between − 15 and − 5 °C. The coalescence of air cells was the dominant coarsening mechanism controlling air bubble size and interconnectivity in the ice cream. Synchrotron X-ray imaging provided sufficient evidence that unfrozen matrix plays role in microstructural stability and the complex interactions between ice crystals and air structures. Similarly, SR X-rays were used with iodine as a contrast agent at 10 keV, 30 keV and 75 keV to measure the three-dimensional distribution of the three main phases (air, unfrozen sugar solution, and ice crystals) of ice cream (Table 1). Quantification of microstructural evolution in ice cream was carried out (Table 1), which revealed that the growth of ice crystals almost ceases after seven thermal cycles, when they approach the size of the walls between air cells, where air cells continue to coarsen, forming interconnected channels [105]

**Fig. 15:**
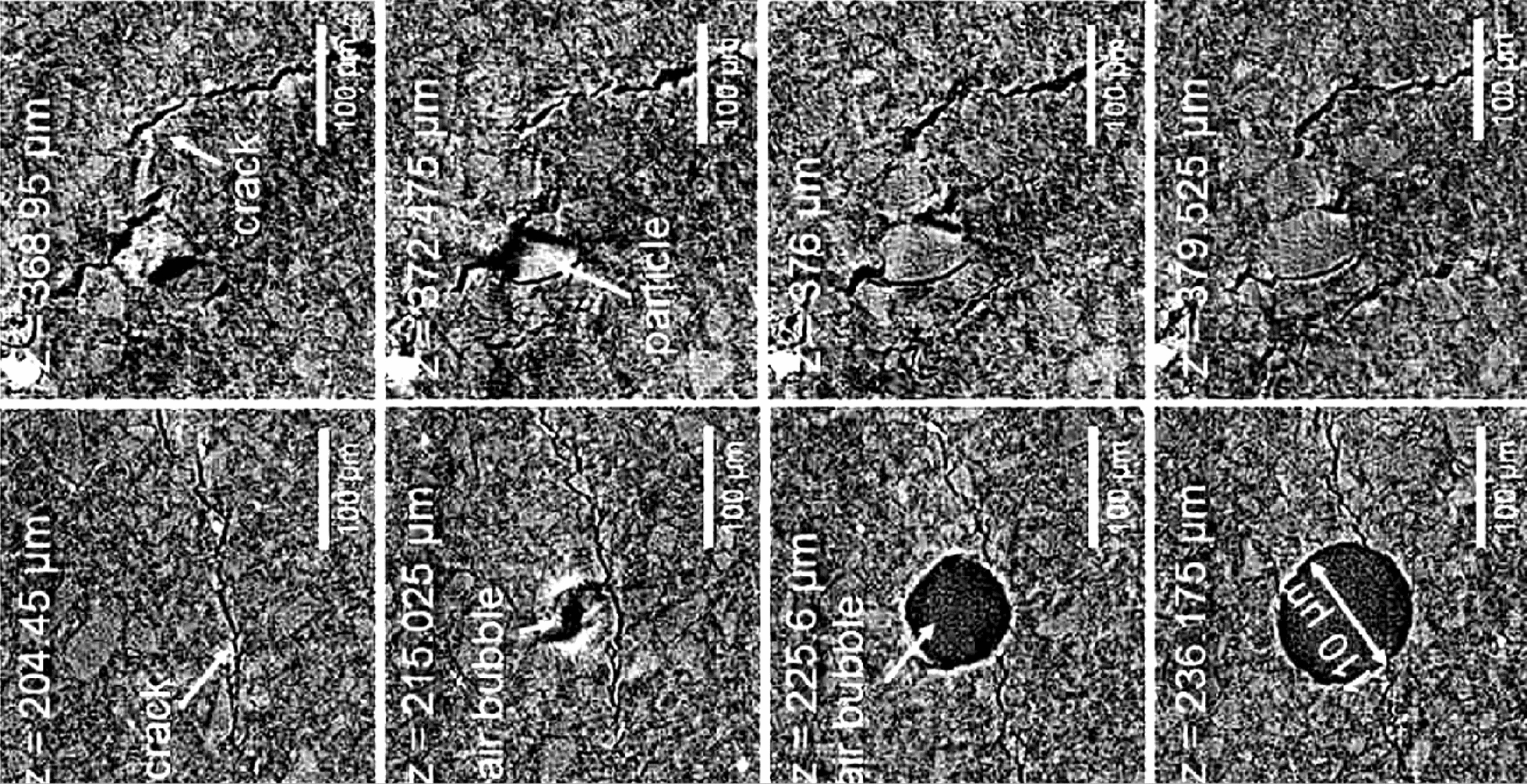
Synchrotron X-ray image slices of different layers showing voids and cracks in chocolate samples [48]

Chocolate blooming is the major problem in the confectionery industry [48] and many studies have been carried out in the past using non-destructive methods, but due to low resolution and high scanning times failed to provide evidence to resolve the blooming issue. Reinke et al. [48] analysed chocolate using SR-μCT (Table 1) and concluded that the convective flow of liquid-lipid fractions in the solid cocoa butter matrix played a significant role as a transport mechanism responsible for fat blooming. This mechanism was validated by the formation of cracks (width of some microns and a length of up to several 100 μm) propagating through the whole chocolate sample (Fig. 15). The imperfections, which arise during the manufacturing process, might act as migration pathways since they propagate throughout the entire chocolate.

Post-harvest storage of perishables is one of the important areas where non-destructive approach is required to study changes during storage. It was reviewed earlier that the current state of the art in modelling tissue microstructures was not sufficient [74] for modelling the 3D configuration of cells, cell walls and intercellular spaces in fruit tissues. Mebatsion et al. [74] applied synchrotron imaging to study post-harvest disorders in pome fruits: apple and pear (Table 1). They have used ANSYS for finite element modelling for quantification and MATLAB programmed algorithms (tessellation algorithm) which yielded smaller apple parenchyma porosity and larger pear parenchyma porosity besides having small sample size. They concluded that multiscale modelling of transport phenomena and mechanical deformation of scanned fruits enhanced the knowledge about fruit environment interactions, and evolution of physiological disorders and can be used to improve cold storage design and control. The connectivity of the voids is essential for mass transfer, and it also determines the tortuosity of the void channels, which are the main pathways for gas exchange. Another research team had generated high-contrast 3D absorption images of in vivo fruit tissues of high moisture content (Table 1), which enabled visualization of individual cell morphology, cell walls, and entire void networks that were previously unknown for understanding spoilage in post-harvest storage [13]. Both reviewed synchrotron methods (phase and absorption contrast imaging) can be used depending on the X-ray energy and sample properties in food product analysis [106].

## Conclusion

The synchrotron X-ray imaging facilities around the world are growing and well established due to their unique properties and application domain. Synchrotron based non-destructive methods enabled researchers engaged in agricultural and food sciences to study structural changes in the material through virtual slicing, as well as on the surface. Phase contrast imaging has great scope for imaging biological materials in harder surrounding matrix, where materials with low X-ray attenuation of interest are located and have differences in refractive index. Researchers were able to find answers to many questions in modern and ancient plants through synchrotron imaging and mapped for the first time the development of seed and fat blooming of chocolate in food science. The study of quick and dynamic processes in plant sciences like absorption of nutrients, and in food science like bubble formation was made possible due to the intervention of synchrotron imaging. The hard SR X-rays were the best for soil medium imaging.

Present studies do not reflect much on the effect of dose on living things (plants and seeds) in terms of germination, viability, the economic feasibility of methods, and tissue damage except a few. The radiation damage in SR imaging is still lower than in conventional systems due to fast imaging and monochromatic beams. SRP-µCT has potential to address problems of tissue damage in SR-µCT, but knowledge of the possible impact of the X-ray dose was not yet assessable. Sample heating and protein denaturation may be caused which may lead to the redistribution of cellular components and experimental artifacts. The reconstruction step requires some prior knowledge of the samples which is accessible in material science but rarely in plant and food science. In SR-µCT high photon fluxes and energy are necessary for high-resolution imaging, but plant tissues may be severely damaged, and this can limit the use of this method in a continuous investigation in plant science. There is a good scope for harnessing synchrotron imaging's full potential using approaches like machine learning and deep learning methods for agriculture applications. The future scope of applications may include in post-harvest management of agriculture produce, standardization of engineering parameters of SR imaging such as energy, like the use of not only high spatial resolution but also high density resolution, optimizing dose and development of computer vision methodology or machine learning algorithms for image processing.

## Data Availability

Not applicable.

## Abbreviations

SR: Synchrotron
SR-μCT: Synchrotron absorption micro computed tomography
SRP-μCT: Synchrotron phase contrast micro computed tomography
SR-XTM: Synchrotron X-ray absorption tomography
HXRTM: High resolution X-ray tomography
SR-PCI: Synchrotron phase contrast imaging
